# Baseline characteristics and hospitalizations in patients with schizophrenia receiving olanzapine long-acting injection: an interim analysis from a non-interventional, prospective observational safety study

**DOI:** 10.1186/s12888-015-0669-5

**Published:** 2015-11-13

**Authors:** Meghan E. Jones, Jeffrey S. Andrews, Douglas E. Faries, John Landry, Jenny Xu, Holland C. Detke, Rashna Chhabra-Khanna, David P. McDonnell

**Affiliations:** Eli Lilly and Company, Indianapolis, IN 46285 USA; Eli Lilly Canada, Inc., Toronto, ON M1N 2E8 Canada; MacroStat (China) Clinical Research Co., Ltd., Shanghai, China; Lilly UK, Windlesham, Surrey GU20 6PH UK; Eli Lilly and Company, Cork, Ireland

**Keywords:** Olanzapine, Long-acting injection, Schizophrenia, Antipsychotics

## Abstract

**Background:**

Depot antipsychotics are a treatment option for medication nonadherence in patients with schizophrenia. Nonadherence can lead to increased relapse and hospitalization rates. This article reports hospitalization data before and after initiation of olanzapine long-acting injection (LAI), a depot antipsychotic.

**Methods:**

Data were assessed from an ongoing, multinational, prospective, observational post-authorisation safety study being conducted to evaluate post-injection delirium/sedation syndrome (PDSS), an adverse reaction that can occur following injection of olanzapine LAI. Eligible patients were aged ≥18 years, diagnosed with schizophrenia, were prescribed olanzapine LAI, and lived outside the United States. Psychiatric hospitalization and medication data were collected retrospectively for the 6-month period before study entry and prospectively throughout the study. Paired t-tests and McNemar’s tests were used to assess changes in hospitalization incidence and duration. Stepwise Cox proportional hazards models assessed factors associated with hospitalizations. Analyses were based on data from the first 3 years of the continuously enrolling study (*N* = 668).

**Results:**

The average duration of olanzapine LAI exposure for all patients was 0.768 years. Of the 529 patients who received at least 1 injection of olanzapine LAI and were not hospitalized at study entry, 8.1 % had at least 1 subsequent psychiatric hospitalization with a mean duration of 2.0 days. Of the 288 patients who had a >6-month follow-up, 8.3 % had at least 1 post-baseline psychiatric hospitalization with a mean duration of 2.3 days. The incidence of hospitalizations in the 6-month period after treatment was significantly lower than that in the 6-month period prior to treatment (8.3 vs 32.6 %, respectively; *P* < 0.001). Furthermore, mean hospitalization duration decreased from 11.5 days in the 6-month period before treatment to 2.3 days in the 6-month period after treatment (*P* < 0.001). Psychiatric hospitalization in the prior 12 months (*P* < 0.0001) and recreational drug use within 24 h of baseline visit (*P* = 0.015) were identified as potential predictors of time to first psychiatric hospitalization after beginning to take olanzapine LAI. At the time of interim analysis, 5 PDSS events had occurred, which was too few for a full analysis of those events.

**Conclusions:**

Results indicate a significant reduction in the incidence and days of hospitalization from the 6-month period before to the 6-month period after olanzapine LAI initiation, which suggests reduced relapse and hospitalization during treatment. Results should be interpreted with caution due to the observational nature of the study and use of retrospective baseline data.

**Electronic supplementary material:**

The online version of this article (doi:10.1186/s12888-015-0669-5) contains supplementary material, which is available to authorized users.

## Background

Schizophrenia is a chronic disease that affects almost 1 % of the world’s population [1]. Nonadherence or partial adherence to drug treatment is relatively common in many chronic illnesses, including schizophrenia [2–6], and the average rate of nonadherence to antipsychotics is approximately 50 % [7]. Medication nonadherence contributes to symptom exacerbation, hospitalization, and relapse [8]. Seventy-five percent of patients who discontinue their medication experience significant exacerbation of symptoms over 1 year compared with 25 % of those who adhere to their medication [9, 10].

Costs associated with treatment received following relapse may comprise the largest segment of treatment costs related to schizophrenia [11–14]. A review of studies addressing the economic impact of antipsychotic nonadherence in individuals with schizophrenia revealed that poor adherence to antipsychotic medications was consistently associated with a higher risk of relapse and rehospitalization, as well as with higher hospitalization costs [15]. Moreover, it has been estimated that most of the direct costs of schizophrenia result from hospitalization or other residential care [15, 16]. Thus, treatments associated with increased adherence and reduced hospitalizations are important for the clinical management of schizophrenia.

Long-acting injectable antipsychotic medications are an important option to help minimize nonadherence because they allow for non-adherent patients to be identified sooner and they remove the need for patients to take medication every day, increasing treatment adherence [17, 18]. Olanzapine long-acting injection (LAI) is a sustained-release intramuscular dosage formulation consisting of a pamoate salt of olanzapine. Olanzapine LAI has been shown to be efficacious for the treatment of schizophrenia in both actively psychotic [19] and stable patients [20]. Using a large claims database, Peng et al. [21] found that psychiatric hospitalizations of patients with schizophrenia who were treated with LAIs decreased from 49.7 % during the 6 months before LAIs initiation to 22.4 % in the 6 months after LAIs initiation.

To assess the impact of olanzapine LAI use on hospitalization, we evaluated the hospitalization rates and durations in the 6 months before and the 6 months after study entry in patients participating in an ongoing multinational observational study. The study was designed with the primary objective of collecting prospective data on post-injection delirium/sedation syndrome (PDSS), an adverse event that has been reported during treatment with this formulation [22, 23]. However, data on patient medication use and hospitalization, historically as well as prospectively, were also collected, providing a unique opportunity to assess the potential impact of olanzapine LAI on outcome measures closely tied to medication adherence. Exploratory analyses were also conducted to assess whether any patient baseline characteristics might be associated with subsequent risk of hospitalization.

## Methods

### Study design

Data were collected as part of a non-interventional, prospective observational post-authorization safety study (PASS) designed to assess the incidence of PDSS events in patients with schizophrenia being treated commercially with olanzapine LAI (see Additional file 1 for STROBE checklist of observational studies) . This report is an interim analysis of the data collected between April 2009 (first patient enrolled) and March 2012 (the first planned interim data cutoff for the study for the purpose of evaluating hospitalization data). This study is ongoing. As of that data cutoff, the study was being conducted in multiple sites in Australia, Austria, Belgium, Czech Republic, Denmark, Finland, France, Germany, Greece, Hungary, Ireland, Italy, Romania, Slovakia, Spain, Sweden, and the United Kingdom.

The study was approved in all countries at the site, regional, or national level, depending on the country and local regulations. Patient also consent followed country-specific regulations. The study protocol was approved by the Ethical Review Board at each study center. Please see Additional file 2 for a full list of participating centers and names of ethics boards.

All patients were receiving commercially available olanzapine LAI in accordance with their physicians’ standard of care. Treatment for schizophrenia with olanzapine LAI was prescribed at the discretion of the investigator and was not provided for or reimbursed by the study sponsor. Throughout the study, effort was made to avoid interference with clinical practice to maintain real-life settings. Investigators were instructed to make treatment decisions independent of the study and then determine whether patients were eligible for study inclusion on the basis of the entry criteria.

### Patients

Eligible patients were men and women aged ≥18 years who had a diagnosis of schizophrenia and whose physicians had prescribed olanzapine LAI. Patients could be enrolled in the study regardless of whether they began treatment with olanzapine LAI prior to the start of the study. Study inclusion was also predicated on the willingness of the patient to sign an informed consent form to release medical information. Patients could not participate if they were investigator site personnel directly affiliated with the study or a member of their immediate families, employees of Eli Lilly and Company, or unwilling to provide written consent to release medical information or other required forms to participate in the study. Patients were not paid for their participation in the study.

### Data collection

Data collection for the study occurred during routine visits within the normal course of healthcare. Baseline data collection occurred at the first administration of olanzapine LAI after patient consent. Data collected at baseline included demographics, year of initial schizophrenia diagnosis, previous antipsychotic medication use, history of alcohol and recreational drug use, and previous psychiatric hospitalization information. Data were also collected at any subsequent office visits during which the patient received an injection of olanzapine LAI. The frequency of injection visits was at the discretion of the investigator. Data collected at every injection visit included adverse events, concomitant medications, and psychiatric hospitalization information.

### Statistical methods

Analyses were applied to all patients who received olanzapine LAI unless otherwise specified. Descriptive statistics were used to describe the study population. For analyses of changes in post-baseline incidence and the duration of psychiatric hospitalization, patients who had exposure to olanzapine LAI in the 6-month period prior to baseline were excluded. McNemar’s test of change was used to compare the proportion of previous psychiatric hospitalizations and the proportion of post-baseline psychiatric hospitalizations among patients with at least 1 injection and without any baseline hospitalizations (defined as patients who were not hospitalized when they initiated treatment with olanzapine LAI). A paired Student’s *t*-test was performed for change in days of hospitalization from previous to post-baseline among patients with at least 1 injection and without baseline hospitalization. Because not all patients currently enrolled in the study had the opportunity for at least 6 months of follow-up, these pre vs. post analyses were performed both to include all of the data and to restrict data to only the population of patients who had the opportunity for 6 months of follow-up (compared to their 6 months prior to baseline). This may result in slightly conservative analyses because the average exposure time in the follow-up period was greater than the 6-month baseline period.

Stepwise (forward) Cox regression was used to identify baseline risk factors associated with time to first psychiatric hospitalization among patients not hospitalized when they initiated olanzapine LAI treatment. Data are presented as hazard ratios (HRs) and 95 % confidence intervals (CIs). The following variables were input as potential variables to be included in the stepwise Cox regression model building process: continuous variables, including age, age at time of first schizophrenia episode, years since first schizophrenic episode, weight, and body mass index; and categorical variables, including gender (male/female), race (white/non-white), recreational drug use within 24 h of receiving first injection of olanzapine LAI (yes/no), alcohol use within 24 h of receiving first injection of olanzapine LAI (yes/no), country (Australia, Austria, Belgium, Czech Republic, Denmark, Finland, France, Germany, Greece, Hungary, Ireland, Italy, Romania, Slovakia, Spain, Sweden, or United Kingdom), antipsychotic treatment during the previous 2 years (yes/no), concomitant antipsychotics (yes/no), other psychiatric medications (yes/no), and psychiatric hospitalization in prior 6 and 12 months (yes/no). The final model included only statistically significant covariates (i.e., those with *P* < 0.10 based on the Wald chi-square test).

## Results

### Baseline patient demographics and clinical characteristics

As of March 31, 2012, at total of 10,085 injections of olanzapine LAI were given to 668 patients in the study. Table 1 shows the demographic and clinical characteristics of the patients at baseline. Patients were mostly male (61.8 %), had a mean age of 40.8 years, had a mean weight of approximately 79.4 kg, and were mostly white (93.3 %). Mean age at onset of schizophrenia was 29.2 years.

**Table 1:** Baseline patient demographics and clinical characteristics of patients receiving olanzapine long-acting injection

Characteristic	All Patients with ≥1 Injection (*N* = 668)
Age (years), mean (SD)	40.8 (11.8)
Gender, *n* (%)	
Male	413 (61.8)
Female	255 (38.2)
Race, *n* (%)	
Black	9 (1.3)
Asian	4 (0.6)
White	623 (93.3)
Missing	32 (4.8)
Weight (kg), mean (SD)	79.4 (17.2)^a^
Age at first episode of schizophrenia (years), mean (SD)	29.2 (10.0)
Psychiatric hospitalization, prior 12 months	
Yes, *n* (%)	321 (48.1)
Number of days (mean, SD)	39.1 (81.6)
Number of days – among only patients with a hospitalization (mean, SD)	81.4 (102.2)
Psychiatric hospitalization, prior 6 months	
Yes, *n* (%)	289 (43.3)
Number of days (mean, SD)	24.3 (46.1)
Number of days – among only patients with a hospitalization (mean, SD)	56.2 (56.0)
Antipsychotic treatment previous 2 years	
Yes, n (%)	590 (88.3)

*Abbreviations*: *N/n* number of patients, *SD* standard deviation

^a^Five patients were missing from the analysis

### Disposition, dosing, and concomitant medication

The average (standard deviation [SD]) duration of olanzapine LAI exposure for all patients was 0.768 (0.69) years, which reflects the ongoing enrollment in the study at the time of the interim analysis. During this time, 31.7 % of patients discontinued the study. The most frequent dose of olanzapine LAI was 300 mg every 2 weeks, which was used for 47.6 % of all injections.

### Hospitalizations

A total of 83 patients already had exposure to olanzapine LAI in the 6 months prior to study entry, leaving 585 patients eligible for post-baseline hospitalization analyses. At baseline, 248 (42.4 %) of 585 patients reported a psychiatric hospitalization in the 6-month period prior to study enrollment, with 273 (46.7 %) patients experiencing a psychiatric hospitalization in the 12-month period prior to enrollment. A total of 56 (10.7 %) patients were still hospitalized at the time of olanzapine LAI initiation and study entry. Across all patients, the mean duration of the psychiatric hospitalization reported was 21.7 days in the 6-month period prior to study enrollment and 33.1 days in the 12-month period prior to enrollment. Among only those patients hospitalized, the mean duration of hospitalization was 51.2 days in the 6 months before study enrollment and 71.0 days in the 12 months before study enrollment. Table 2 summarizes the rate and duration of post-baseline psychiatric hospitalizations at initiation of olanzapine LAI treatment. Of the 529 patients who were not hospitalized at study entry and received at least 1 injection of olanzapine LAI, 43 (8.1 %) had at least 1 subsequent psychiatric hospitalization, with a mean duration of 2.0 days. Of the 288 patients who had >6-months follow-up, 24 (8.3 %) had at least 1 post-baseline psychiatric hospitalization with a mean duration of 2.3 days.

**Table 2:** Rate and duration of post-baseline psychiatric hospitalizations^a^

Baseline Hospitalization^b^	Characteristic	Patients with ≥1 Olanzapine LAI Injection	Patients with Opportunity for >6-months Follow-up
No	N	529	288
	Patients with ≥1 post-baseline hospitalization, n (%)	43 (8.1)	24 (8.3)
	Days of hospitalization, mean (SD)	2.0 (12.0)	2.3 (14.3)
	Days of hospitalizations among patients with a hospitalization, mean (SD)	24.9 (35.0)	27.0 (43.1)
Yes	N	56	29
	Patients with ≥1 post-baseline hospitalization, *n* (%)	6 (10.7)	5 (17.2)
	Days of hospitalization, mean (SD)	3.6 (20.5)	6.8 (28.3)
	Days of hospitalizations among patients with a hospitalization, mean (SD)	33.3 (58.5)	39.4 (63.3)

*Abbreviations*: *LAI* long-acting injection, *N/n* number of patients, *SD* standard deviation

^a^Analysis included only patients who started treatment with olanzapine LAI at time of study entry

^b^Defined as hospitalization upon initiation of olanzapine LAI treatment

Table 3 demonstrates that the incidence of hospitalizations after olanzapine LAI treatment was statistically significantly lower than the incidence of hospitalizations in the 6-month period prior to olanzapine LAI treatment (*P* < 0.001). In addition, there was a significant reduction in the number of days hospitalized; mean hospitalization duration was 11.5 days in the prior 6 months and decreased to 2.3 days in the post-baseline period (*P* < 0.001). These pre vs. post analyses included only those patients with opportunity for at least 6-months follow-up. Analyses using data from all patients were consistent in demonstrating significant reductions in the incidence of hospitalization and the number of days hospitalized.

**Table 3:** McNemar’s test of pre- versus post-baseline hospitalizations^a^

	Post-baseline Hospitalization		
Previous Hospitalization^b^	No	Yes	*P*-value
No, *n* (%)	183 (63.5)	11 (3.8)	<0.001
Yes, *n* (%)	81 (28.1)	13 (4.5)	

*Abbreviation*: *LAI* long-acting injection, *SD* standard deviation

^a^Analysis included only patients who started treatment with at least 1 injection of olanzapine LAI at time of study entry, without baseline hospitalization, and who had opportunity for at least 6 months of follow-up

^b^Analysis included hospitalization in the 6 months prior to initiating olanzapine LAI treatment

**Fig. 1:**
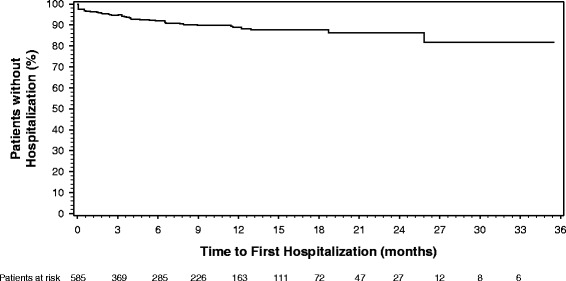
Time to first hospitalization for patients treated with olanzapine long-acting injection at study entry. Hospitalization rates were low over the course of treatment, with an estimate of about 80 % of patients remaining unhospitalized after 36 months

Figure 1 shows the Kaplan-Meier curve of time to hospitalization in the 36 months after the initiation of olanzapine LAI treatment. Hospitalization rates were low over the course of treatment, with approximately 80 % of patients remaining unhospitalized after 36 months.

Table 4 summarizes the risk factors associated with time to first psychiatric hospitalization that were identified using a stepwise Cox regression approach. Among all of the variables used in the model, the strongest association identified was that patients who were hospitalized in the previous 12 months for psychiatric reasons were more likely to be hospitalized during the study (HR = 5.00, *P* < 0.0001). Recreational drug use within 24 h of the baseline study visit (HR = 5.90, *P* = 0.015) was also identified as a potential risk factor associated with time to first psychiatric hospitalization, although the small sample size and large CI limits are noted.

**Table 4:** Risk factors for time to first psychiatric hospitalization^a^

		95 % Confidence Interval		
Variable	Hazard Ratio	Lower	Upper	*P*-value^b^
Psychiatric hospitalization in prior 12 months	5.00	2.41	10.37	<0.0001
Recreational drug use within 24 h of baseline study visit	5.90	1.41	24.59	0.015

^a^Analysis included only patients who started treatment with olanzapine LAI at time of study entry

^b^Cox regression model was applied

### Post-injection delirium/sedation syndrome

Five patients experienced 5 adverse events that were determined to be PDSS events. As of March 31, 2012, based on 10,085 injections of olanzapine LAI given to 668 patients, PDSS events occurred in approximately 0.05 % of injections, or 0.75 % of patients. Due to the small number of PDSS events, analyses on PDSS risk factors could not be conducted as part of this interim analysis and will be conducted on final data lock (after achieving the protocol-specified target of 92,500 injections).

## Discussion

Although the primary purpose of this ongoing observational study is to conduct a post-marketing evaluation of PDSS in patients with schizophrenia treated with olanzapine LAI, interim data analysis after the first 3 years of data collection allowed for the examination of patient baseline characteristics and rates of psychiatric hospitalization before and after initiation of olanzapine LAI. Psychiatric hospitalization is an important clinical outcome that carries a high personal and economic burden. It can also serve as an indicator of patient nonadherence and is relevant when evaluating a depot medication that is meant to address nonadherence. Interim analysis of the current study found that patients receiving >6 months of olanzapine LAI in the post-baseline period had significantly lower rates of psychiatric hospitalization than during the 6 months before study baseline (incidence: 8.3 % versus 32.6 %, respectively; *P* < 0.001), as well as reduced hospitalized days (mean [SD] days: 11.5 [23.8] versus 2.3 [14.3]; *P* < 0.001). Patients with exposure to olanzapine LAI in the 6 months prior to study entry were excluded from the hospitalization analyses to reduce any potential effect of prior exposure of olanzapine LAI on the outcome of hospitalization.

The results related to hospitalization data in this study are consistent with those reported previously in literature. In a recent study in previously nonadherent patients, those receiving depot typical antipsychotics versus oral typical antipsychotics reported better medication continuation, decreased hospitalization rates, and decreased mean number of hospitalizations [24]. Similarly, a study in a home health care setting in Taiwan showed a significantly lower risk for psychiatric rehospitalization in patients receiving LAI antipsychotics than those treated with oral medication [25]. In addition, a United States Medicaid database study reported that use of LAI medications is associated with a longer time to the first hospitalization for any cause and for schizophrenia; moreover, the authors also reported that a longer use duration of LAI antipsychotics (at least 6 months relative to those treated for shorter durations) is associated with a decreased number of hospitalizations, decreased hospital length of stay, and reduced hospital payment—thus using fewer hospital resources [26]. Findings from randomized clinical trials of olanzapine LAI have also produced similar results indicating favorable changes in hospitalization [27].

Across all patients in the current study, Kaplan-Meier estimate indicated that approximately 80 % of patients remained free of psychiatric hospitalization during the 3-year data collection period. Time to first psychiatric hospitalization was best predicted by psychiatric hospitalization in the 12 months prior to starting olanzapine LAI. This finding is consistent with previous research that has shown past hospitalizations to be a strong predictor of future hospitalizations [27–30]. In addition, recreational drug use within 24 h of receiving the first injection of olanzapine LAI was identified as a potential baseline correlate of time to first psychiatric hospitalization. However, caution should be taken when interpreting the individual contributions of each factor due to the small subgroup size and the potential correlation among the variables in the final model.

At the time of the interim analysis, 5 PDSS events had occurred in 5 patients in the trial, indicating an incidence of 0.05 % of injections, or approximately 1 event per 2017 injections. Although the incidence and nature of the events were similar to those described previously from the olanzapine LAI clinical trials [19, 22], too few events occurred to obtain a detailed meaningful analysis at this time. Furthermore, the average duration of olanzapine LAI exposure for all patients was 0.768 years in this interim analysis. Final study results will provide a better estimate of exposure.

Certain limitations to this study must be noted. First, because an observational study design was used, patients were not randomized to treatment and the results may have been affected by selection bias. Although the strength of an observational design is its ability to directly observe individuals in their natural setting and to better reflect clinical practice, the resulting populations of patients (and investigators) who elect to participate in the study may be determined by individual preferences, practice patterns, or policy decisions [31]. It also should be noted that data collected at baseline regarding previous hospitalizations and medications represent retrospective data and are subject to limitations in recall or reporting. In addition, the recall period for reporting alcohol and recreational drug use was limited to the 24 h prior to receiving the first injection of olanzapine LAI and may not fully characterize the history and extent of consumption by study participants. Caution should be used when interpreting the association between acute use of these predictor variables and psychiatric hospitalizations. Adequate numbers were not available to analyze 12-month post study enrollment hospitalization data, so these data were not included in this report. Furthermore, the types and amount of data collected in an observational study are more limited than would be the case in a controlled clinical trial; data collection was kept to a minimum to not burden investigators and patients and to not interfere with typical clinical practice.

## Conclusions

The 6-month results indicate a significant reduction in the incidence and days of hospitalization from the period before to the period after olanzapine LAI initiation. The current results support findings in the published literature that depot antipsychotics reduce relapse and hospitalization. Psychiatric hospitalization in the previous 12 months and recreational drug use in the 24 h prior to baseline study visit were identified as potential predictors of time to first psychiatric hospitalization. Because findings were from an interim analysis, final study results will be necessary to determine whether the current findings are maintained and to elaborate further on clinical presentation, outcomes, and potential risk factors associated with PDSS.

### Availability of supporting data

Additional information about the dataset and analyses are available upon request, but the data files are the proprietary property of Eli Lilly and Company.

## Additional files

Additional file 1:
**A STROBE checklist for observational studies.** (PDF 22 kb) Additional file 2:
**A list of participating centers and names of ethics boards.** (XLS 71 kb)

## Abbreviations

CI: Confidence interval
HR: Hazard ratio
LAI: Long-acting injection
PASS: Post-authorisation safety study
PDSS: Post-injection delirium/sedation syndrome
SD: Standard deviation
